# Interpretable deep learning for functional MRI-based auxiliary diagnosis of major depressive disorder with suicidal ideation

**DOI:** 10.3389/fncom.2026.1821002

**Published:** 2026-07-07

**Authors:** Xiao Li, Xiangyu Chen, Xinge Du, Shaoyong Guo, Junfeng Ma, Ting Pang

**Affiliations:** 1 School of Medical Engineering, Henan Medical University, Xinxiang, China; 2 Xinxiang High Performance Computing Medical Engineering Technology Research Center, Xinxiang, China

**Keywords:** BrainNet convolutional neural network, diagnosis of depression with suicidal ideation, functional magnetic resonance imaging, interpretable deep learning, salient brain region features

## Abstract

**Introduction:**

Suicidal ideation (SI) in patients with major depressive disorder (MDD) is frequently underrecognized in early clinical assessment, owing to the complexity of its underlying neurobiological mechanisms and the lack of complementary objective biomarkers. To address this issue, this study proposes an interpretable deep learning framework designed to assist in the diagnosis of patients with MDD with suicidal ideation (MDDSI) and to elucidate its underlying neural mechanisms.

**Methods:**

A modified BrainNet convolutional neural network architecture was employed, utilizing a whole-brain functional connectivity (FC) matrix derived from resting-state functional magnetic resonance imaging as input features. A gradient-weighted class activation mapping algorithm was subsequently applied to visualize key brain regions. The study included 356 patients with MDDSI and 107 patients with MDD without suicidal ideation (MDDNSI) as the control group.

**Results:**

Five-fold cross-validation indicated that the model achieved an accuracy of 88%, sensitivity of 95%, specificity of 85%, and an area under the curve of 0.93 on the test set. Feature visualization results revealed that the model’s classification decisions primarily relied on abnormal FC patterns in regions such as the motor cortex, anterior cingulate cortex, occipital lobe, temporal lobe, parietal lobe, and cerebellum.

**Discussion:**

This work provides a valuable reference for both auxiliary diagnosis and the mechanistic investigation of MDDSI.

## 1 Introduction

Major depressive disorder (MDD) is a prevalent psychiatric condition characterized by a persistently depressed mood and diminished interest or pleasure in activities (Monroe and Harkness, 2022; Whiteford et al., 2013). According to the World Health Organization, the lifetime prevalence of depression reaches approximately 16%, affecting more than 350 million individuals globally (Liu et al., 2020), underscoring its considerable public health burden. Importantly, the risk of suicidal behavior among individuals with MDD is estimated to be nearly 20 times higher than that in the general population (Borentain et al., 2020; Cai et al., 2021; Nock et al., 2009). Epidemiological studies suggest that 50–70% of patients with MDD experience suicidal ideation (SI) (World Health Organization, 2017). Therefore, SI serves as a critical clinical warning indicator in MDD, and its early detection is essential for timely intervention and for reducing the risk of subsequent suicidal behaviors.

Functional Magnetic Resonance Imaging (fMRI) is a technique capable of quantitatively assessing impaired brain function in mental disorders. Mounting research evidence indicates that the functional connectomes of patients with MDDSI exhibit abnormalities. For instance, Li et al. investigated functional connectivity (FC) patterns in patients with major depressive disorder and found that those with suicidal ideation exhibited diminished FC between the hippocampus and both the superior frontal gyrus and anterior cingulate cortex compared to non-SI patients (Li et al., 2022). These findings suggest that the hippocampus may serve as a critical structural and functional substrate for the emergence of suicidal ideation within the MDD population. Furthermore, Yang et al. conducted an amygdala-based FC analysis across with MDD with suicidal ideation (MDDSI), MDD without suicidal ideation (MDDNSI), and healthy controls (HCs), revealing aberrant connectivity between the lateral amygdala and the caudate nucleus, middle temporal gyrus, and postcentral gyrus in the MDDSI group (Yang et al., 2022). Consequently, they proposed that the aberrant integrity of the cortico-limbic-striatal circuit centered on the amygdala provides a promising neural substrate for suicidal ideation. However, these studies primarily rely on traditional group-level comparative analyses, which struggle to account for the inherent clinical heterogeneity among psychiatric patients and thus limit their utility in individualized clinical assessments.

In this context, the integration of deep learning and connectomics facilitates auxiliary diagnosis at the individual level while providing objective evidence for understanding the neural mechanisms of MDDSI (Gao et al., 2023; Guo et al., 2025). By leveraging dynamic functional network connectivity (dFNC) analysis, prior research has demonstrated the utility of functional connectivity patterns in identifying suicide risk among MDD populations. Specifically, after partitioning windowed dFNC data into discrete states using k-means clustering, an SVM-based classification between patients with and without suicidal ideation yielded a robust AUC of 0.82 (Identification of suicidality in patients with major depressive disorder via dynamic functional network connectivity signatures and machine learning). In contrast to machine learning approaches that often rely on pre-defined, hand-crafted features, Deep Learning architectures offer a transformative paradigm for modeling the high-dimensional complexity of the human connectome. Recent advancements, such as the BrainGB benchmark framework (Cui et al., 2023), have standardized the application of Graph Neural Networks (GNNs) in brain network analysis, demonstrating their superior ability to preserve the brain’s intrinsic spatial topology through message-passing mechanisms (Lee et al., 2025). Furthermore, the integration of Transformer-based models has enabled the capture of long-range functional dependencies and global network reconfigurations, which are often overlooked by local graph metrics (Zhu et al., 2025). However, the existing literature on predicting suicidal ideation in MDD patients remains predominantly rooted in traditional machine learning frameworks, and the potential of deep learning has yet to be systematically exploited in this domain.

To address these limitations, we developed an interpretable deep learning framework based on fMRI to enable objective identification of MDDSI. Specifically, the BrainNet CNN architecture was adapted to model whole-brain FC matrices, and Gradient-weighted Class Activation Mapping (Grad-CAM) was incorporated to generate visual explanations of salient neural features. This framework is designed not only to enhance diagnostic support for MDDSI at the individual level but also to localize potential connectivity-based neuroimaging biomarkers by highlighting discriminative functional connections. Furthermore, it aims to identify specific patterns of FC associated with MDDSI, thereby providing neuroimaging-based evidence to support the understanding of its underlying neurobiological mechanisms.

## 2 Materials and methods

**Figure 1:**
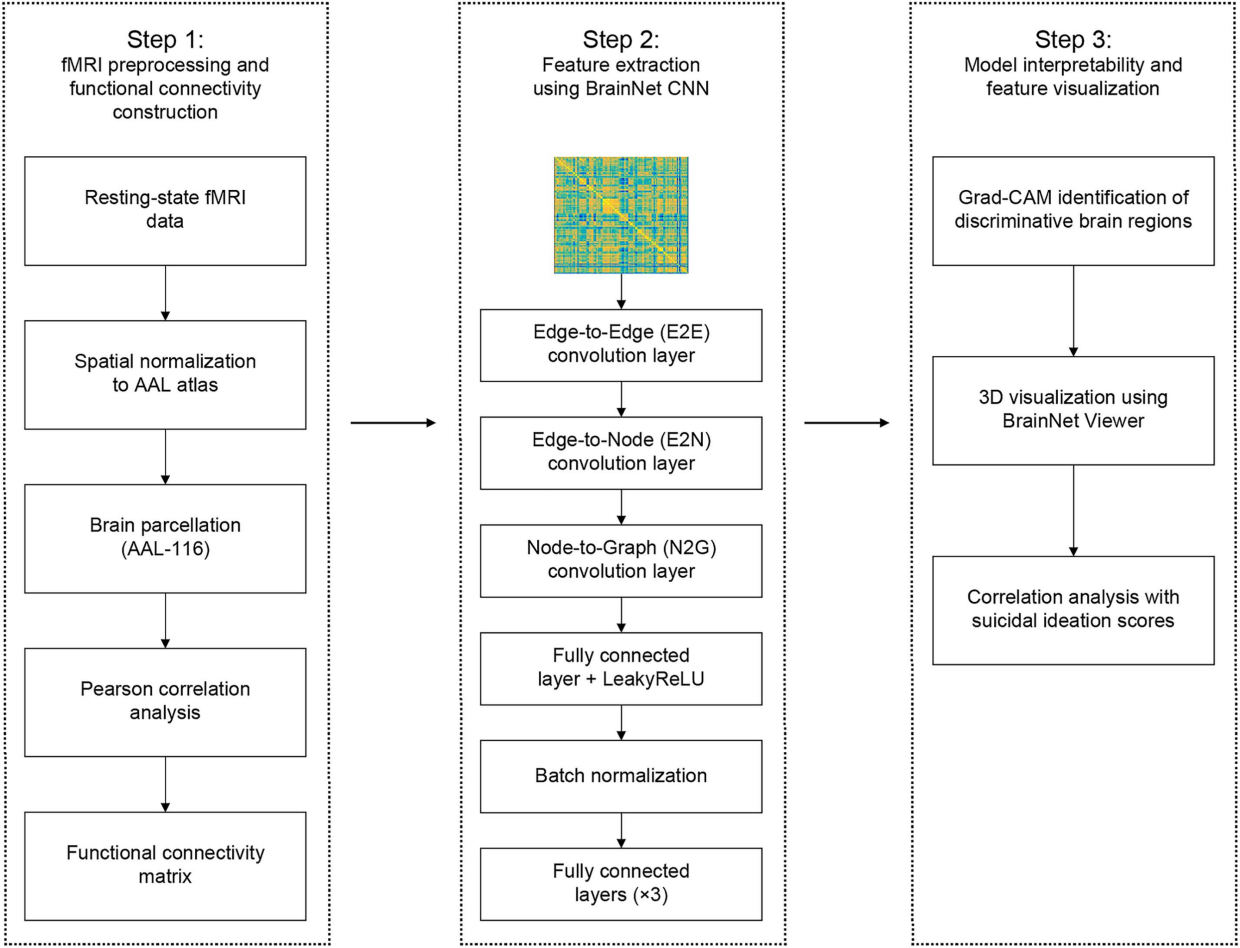
Overview of the proposed framework.

The proposed deep learning-based diagnostic framework for MDDSI was implemented in three sequential stages. First, fMRI data were preprocessed, and whole-brain FC matrices were generated for all of the participants. Second, discriminative features were extracted from individual FC matrices using the BrainNet CNN architecture, which were then utilized for MDDSI classification. Third, the Grad-CAM algorithm was applied to identify brain regions and FCs that contributed most significantly to the model’s classification decisions. In addition, correlation analyses were conducted to assess the relationships between the identified differential FC patterns and SI scores derived from the Hamilton Depression Rating Scale (HAMD). An overview of the proposed framework is provided in Figure 1.

*Step 1*: Preprocessing of resting-state fMRI data and construction of whole-brain FC matrices. *Step 2*: Feature extraction and classification using the BrainNet CNN architecture based on functional connectivity. *Step 3*: Model interpretability analysis, including identification and visualization of discriminative brain regions.

### 2.1 Data acquisition

This study utilized data from the publicly available Rest-meta-MDD consortium (Yan et al., 2019), which compiles 25 openly shared fMRI datasets collected across 17 hospitals.^1^ The dataset includes fMRI scans from 1,300 patients with MDD and 1,128 healthy controls. Detailed acquisition parameters for each center are listed in Table 1. In addition to imaging data, each dataset includes demographic and clinical variables such as age, sex, illness duration, medication status, episode type (first-episode vs. recurrent), and total scores on the HAMD-17 (Williams, 1988). All of the MDD patients met diagnostic criteria defined by either the International Classification of Diseases, Tenth Revision (ICD-10), or the Diagnostic and Statistical Manual of Mental Disorders, Fourth Edition (DSM-IV). Written informed consent was obtained from all of the participants in accordance with protocols approved by the institutional review boards of the contributing centers.

**Table 1:** Scanner models and acquisition parameters across participating sites.

Site	Scanner	Field of view	TR (ms)	TE (ms)	Flip angle (°)	Thickness (mm)	Gap (mm)	No. of axial slices	No. of volumes	Voxel size (mm^3^)
1	Siemens Tim Trio (3T)	210 × 210	2000	30	90	4	0.8	30	210	3.28 × 3.28 × 4.8
2	Philips Achieva (3T)	240 × 240	2000	30	90	4	**—**	37	200	1.67 × 1.67 × 4
6	Siemens Tim Trio (3T)	230 × 230	2000	30	70	4	**—**	33	180	3.59 × 3.59 × 4
7	GE discovery MR750	220 × 220	2000	30	90	3.2	**—**	37	184	2.29 × 2.29 × 3.2
8	GE Sigma (3T)	240 × 240	2000	30	90	3	**—**	35	200	3.75 × 3.75 × 3
11	GE Sigma (3T)	240 × 240	2000	30	90	5	**—**	33	200	3.75 × 3.75 × 5
12	GE Sigma (3T)	240 × 240	2000	30	90	5	**—**	33	240	3.75 × 3.75 × 4
14	Siemens Tim Trio (3T)	240 × 240	2,500	25	90	3.5	**—**	39	200	3.75 × 3.75 × 3.5
18	Philips Achieva (3T)	240 × 240	2000	35	90	5	1.0	24	200	1.67 × 1.67 × 6
19	GE Sigma (3T)	220 × 220	2000	22.5	30	4	0.6	33	240	3.44 × 3.44 × 4.6
21	Siemens Tim Trio (3T)	200 × 200	2000	30	90	3.5	0.7	33	240	3.12 × 3.12 × 4.2
22	Philips Gyroscan Achieva (3T)	240 × 240	2000	30	90	4	**—**	36	250	1.67 × 1.67 × 4
23	Philips Achieva (3T TX)	240 × 240	2000	30	90	4	**—**	38	240	3.75 × 3.75 × 4

**Figure 2:**
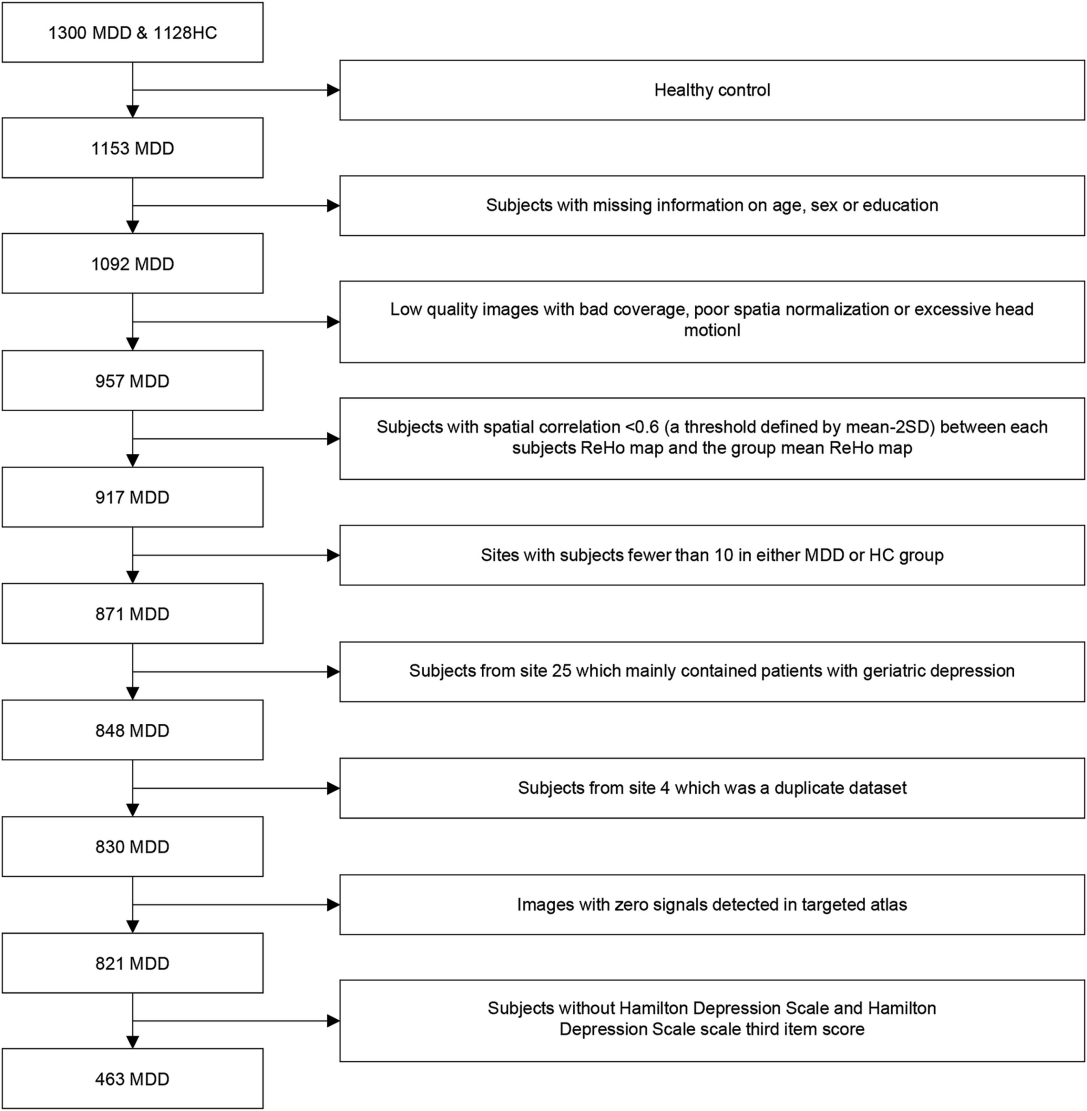
Data screening flowchart.

In accordance with the standardized quality control procedures established by the Rest-meta-MDD consortium, datasets of insufficient quality were excluded based on predefined criteria, as detailed in previous research and illustrated in Figure 2 (Yan et al., 2019). Following this, MDD patients were stratified based on Item 3 (“suicidal ideation”) of the HAMD-17. Participants with scores from 1 to 4 on Item 3 were categorized as having MDDSI, while those with a score of 0 were categorized as MDDNSI (see Figure 2 for further details). After applying the quality control and subgrouping criteria, a total of 463 patients were retained for final analysis, including 356 in the MDDSI group and 107 in the MDDNSI group.

All participants in the original studies provided written informed consent, and data collection at each site within the Rest-meta-MDD consortium was approved by the respective local Institutional Review Boards. The present study is a secondary analysis of these deidentified and anonymized datasets.

### 2.2 Data preprocessing and FC matrix construction

Following the acquisition of resting-state fMRI and three-dimensional T1-weighted MRI data at each participating site, all of the functional images were preprocessed in a standardized manner using the Data Processing Assistant for Resting-State fMRI toolbox (Yan and Zang, 2010). The preprocessing pipeline included the following steps: (1) removal of the first 10 volumes to allow for signal equilibration; (2) slice-timing correction and rigid-body head motion realignment; (3) coregistration of functional images to individual T1-weighted images, followed by spatial normalization to the Montreal Neurological Institute standard space using the Diffeomorphic Anatomical Registration Through Exponentiated Lie Algebra algorithm; (4) spatial smoothing using a Gaussian kernel with 4 mm full-width at half-maximum; and (5) temporal band-pass filtering within the frequency range of 0.01–0.1 Hz. To mitigate the influence of non-neuronal confounds, nuisance signal regression was conducted using the Friston-24 head motion parameters, along with mean signals from the global brain, white matter, and cerebrospinal fluid compartments. Additionally, motion-related artifacts were controlled using a scrubbing procedure with a stringent framewise displacement (FD) threshold of 0.2 mm, whereby volumes exceeding this threshold were excluded. During subject-level quality control, participants with a mean FD greater than 0.2 mm were removed to ensure data quality.

**Figure 3:**
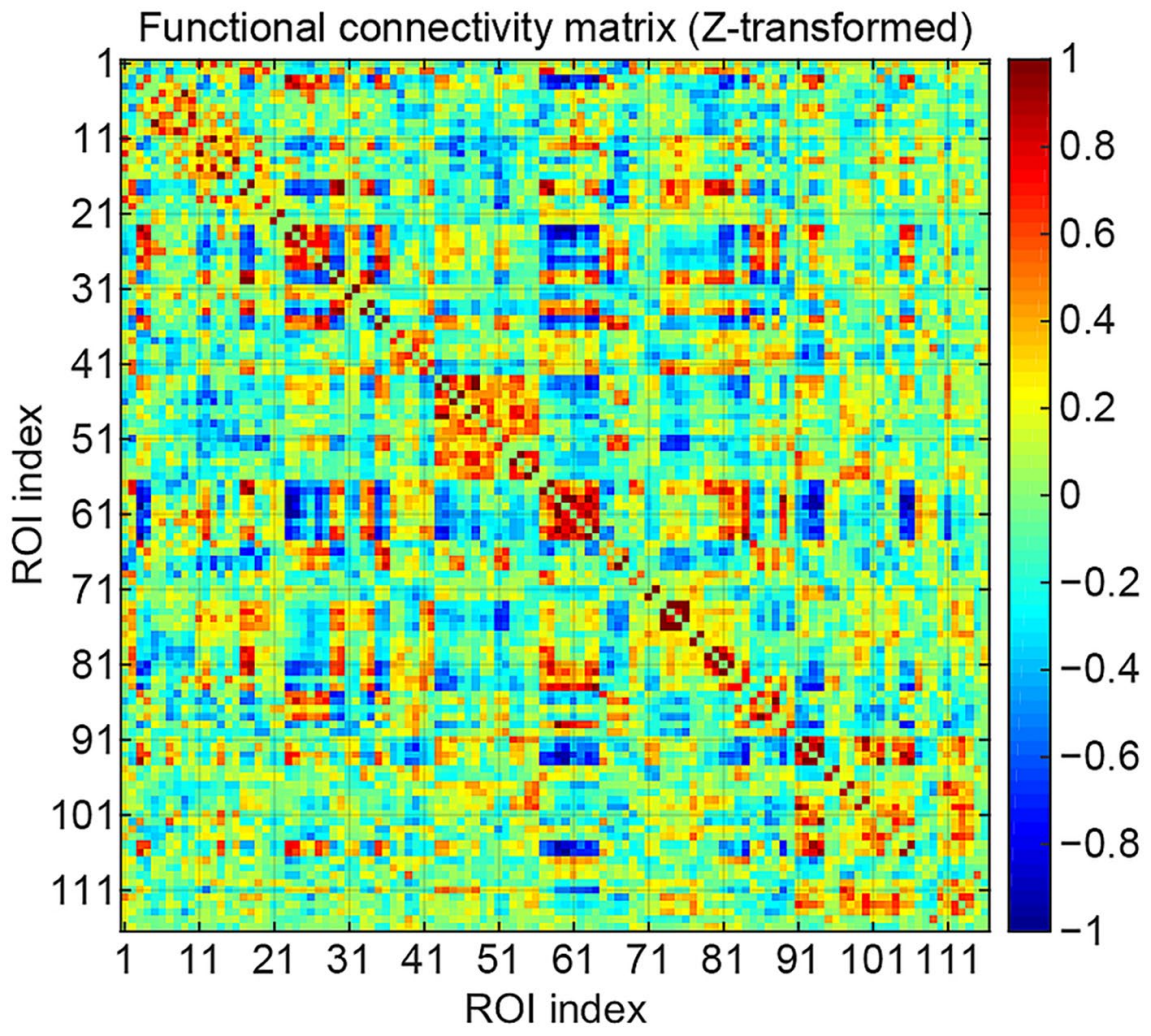
Schematic diagram of FC matrix.

Following preprocessing, whole-brain parcellation was performed using the Automated Anatomical Labeling atlas comprising 116 regions (AAL-116) (Tzourio-Mazoyer et al., 2002). For each participant, blood oxygenation level–dependent time series were extracted by averaging the signals across all of the voxels within each region of interest (ROI). FC between brain regions was quantified by calculating Pearson correlation coefficients between the mean time series of all of the ROI pairs. These correlation coefficients were then transformed to Fisher’s z values to improve normality, and diagonal elements were set to zero, resulting in a symmetric 116 × 116 FC matrix for each participant (Figure 3).

Following the aforementioned preprocessing pipeline, the overall distribution of functional connectivity values for all participants was systematically characterized. Results indicated that connectivity strengths were symmetrically distributed around zero, with negative correlations comprising 52.4% of all of the FCs. Most connections exhibited weak to moderate strength. Network sparsity analyses further revealed a stable inverse relationship between the proportions of positive and negative connections across a broad range of sparsity thresholds, a pattern that remained consistent across all levels. Additionally, threshold sensitivity analyses showed that group differences in the proportion of negative connections were robust to variations in threshold selection, indicating that the observed effects were not driven by specific sparsity thresholds.

### 2.3 Feature extraction using the BrainNet CNN architecture

In this study, a modified BrainNet CNN architecture, originally proposed by Kawahara et al. (2017), was employed to analyze FC matrices for MDDSI identification. FC strength between brain regions was quantified using Pearson correlation coefficients, which were represented as weighted edges in the FC matrices. BrainNet CNN is a convolutional neural network specifically designed to learn topological representations of brain connectivity data. Unlike conventional CNNs used for image analysis, which apply spatially localized filters, BrainNet CNN incorporates specialized convolutional filters that respect the non-Euclidean structure and topological properties of brain networks. This architectural design addresses the fundamental differences between volumetric images and connectivity matrices, thereby enabling effective extraction of discriminative network-level features and making it particularly suitable for modeling functional brain connectomes.

The BrainNet CNN architecture integrates three specialized layer types: edge-to-edge (E2E), edge-to-node (E2N), and node-to-graph (N2G) layers. Each layer is designed to capture distinct topological features of brain networks through convolutional filters specifically tailored for connectivity matrices. Building upon the original BrainNet CNN framework, several modifications were introduced to enhance training efficiency and generalization performance, thereby improving the model’s applicability to real-world scenarios. Specifically, a dynamic input-dimension adaptation mechanism was implemented to accommodate heterogeneity in brain network data derived from multiple sources. Additionally, batch normalization was applied to the fully connected layers to accelerate convergence, stabilize training, and reduce internal covariate shift. An overview of the modified BrainNet CNN architecture is illustrated in Step 2 of Figure 1.

#### 2.3.1 E2E layer

The E2E filters are defined based on the topological relationships between connections, such that this layer functions as a topological pattern recognizer, capturing spatially structured dependencies inherent in the brain network architecture. By aggregating weights of edges that share common nodes, it identifies high-order relationships between functional connections. Intuitively, it captures how clusters of brain regions co-activate, allowing the model to move beyond simple pairwise correlations to understand the “neighborhood” structure of the brain network. Since the number of nodes remains constant between the input and output of this layer, the dimensionality of the adjacency matrix produced by the E2E layer is preserved, and is formally defined as follows:


$$A_{i,j}^{l+1,n}=\sum_{m=1}^{M^{l}}\sum_{k=1}^{|\Omega |}r_{k}^{l,m,n}A_{i,k}^{l,m}+c_{k}^{l,m,n}A_{k,j}^{l,m}$$

where $\left[c^{l,m,n},r^{l,m,n}]=w^{l,m,n}\in R^{2\Omega },\text{and}\;[w^{l,1,n},\ldots ,w^{l,M^{l},n}\right]$ $\in R^{2{|\Omega |}^{\times }M^{l}}$ represents the learned weights of the (n)-th filter in the (l)-th layer. The resulting output $A^{l+1,n}$ is a matrix in $R^{\Omega \times \Omega }$ .

#### 2.3.2 E2N layer

The E2N layer serves as a critical transitional module that bridges local edge-level features with global node-level representations. Its primary function is to adaptively aggregate connectivity information extracted from network edges by the preceding E2E layer into compact functional representations of individual brain regions. By integrating features from the neighboring edges of each node, the E2N layer constructs node-level feature vectors that encapsulate local connectivity patterns and regional interaction profiles. These semantically enriched intermediate representations provide a robust foundation for subsequent modeling stages, enabling the effective integration of network-wide information in downstream layers of the architecture.

The E2N layer receives an adjacency matrix $A^{l,m}\;$ from each feature map as input and outputs a vector of size ∣Ω∣ , defined as follows:


$$a_{i}^{l+1,n}=\sum_{m=1}^{M^{l}}\sum_{k=1}^{|\Omega |}r_{k}^{l,m,n}A_{i,k}^{l,m}+c_{k}^{l,m,n}A_{k,i}^{l,m}$$

where $\left[c^{l,m,n},r^{l,m,n}]=w^{l,m,n}\in R^{2\Omega },\text{and}\;\;[w^{l,1,n},\ldots ,w^{l,M^{l},n}\right]$ , and $\in R^{2{|\Omega |}^{\times }M^{l}}$ denotes the learned weights of the (n)-th filter in the (l)-th layer. The output $a^{l+1,n}$ is a vector in $R^{\Omega \times 1}$ .

#### 2.3.3 N2G layer

The N2G layer performs a weighted combination of node features to generate a single scalar output, thereby reducing the dimensionality of the input. This operation can be interpreted as an aggregation of responses from all of the nodes within the graph to extract global-level features. It is formally defined as follows:


$$a^{l+1,n}=\sum_{m=1}^{M^{l}}\sum_{i=1}^{|\Omega |}w_{i}^{l,m,n}a_{i}^{l,m}$$

N2G filters are typically applied following the E2N filters. The E2N layer transforms adjacency matrices into node-level vectors, and the subsequent N2G layer further processes these vectors by reducing the spatial dimension of each feature map to a single scalar. By aggregating node-level responses from the E2N layer, the N2G layer generates a unified representation that captures global, graph-level information, thereby achieving further dimensionality reduction.

### 2.4 Model interpretability and feature visualization

Grad-CAM (Selvaraju et al., 2019) is a gradient-based visualization technique that quantifies the contribution of intermediate feature maps to a model’s prediction by computing the gradients of the target class with respect to network activations. In this study, Grad-CAM was employed to identify brain regions that contributed most significantly to the classification of MDDSI. Specifically, for a selected convolutional layer, gradients of the target class logit with respect to the channel-wise feature maps were computed and globally average-pooled over spatial dimensions to obtain the importance weights for each channel. These weights were then used to linearly combine the activation maps of the convolutional layer, followed by the application of a rectified linear unit to retain only the features that contributed positively to the target class, thereby generating a class-discriminative activation heatmap.

Within the BrainNet CNN architecture, the second E2E convolutional layer was selected as the target layer for Grad-CAM analysis. This intermediate layer operates directly on edge-level topological representations of the FC matrix, thus capturing discriminative connectivity patterns before higher-level abstraction and integration. To enhance the robustness and reproducibility of the identified key brain regions, a multi-seed validation strategy was adopted. Four independent random seeds were used to perform five-fold cross-validation, resulting in 20 training iterations in total. For each iteration, the best-performing model was selected to compute an average Grad-CAM contribution map across all of the samples.

Brain regions were subsequently ranked according to their contribution scores, and the top 30 regions were selected as candidate key regions. The frequency of each region’s occurrence across all of the models was then calculated. Regions appearing in at least 10 of the 20 trained models were considered to be most strongly associated with MDDSI. To obtain unit-free and comparable contribution metrics, individual-level contribution vectors were normalized such that the sum of contributions across all 116 regions equaled 1, yielding relative contribution values for each participant. Group-level regional importance was calculated by averaging these normalized contributions across all of the subjects. Finally, the BrainNet Viewer toolbox in MATLAB (Xia et al., 2013) was used to generate multi-angle visualizations of the identified key brain regions.

### 2.5 Model training and evaluation

**Figure 4:**
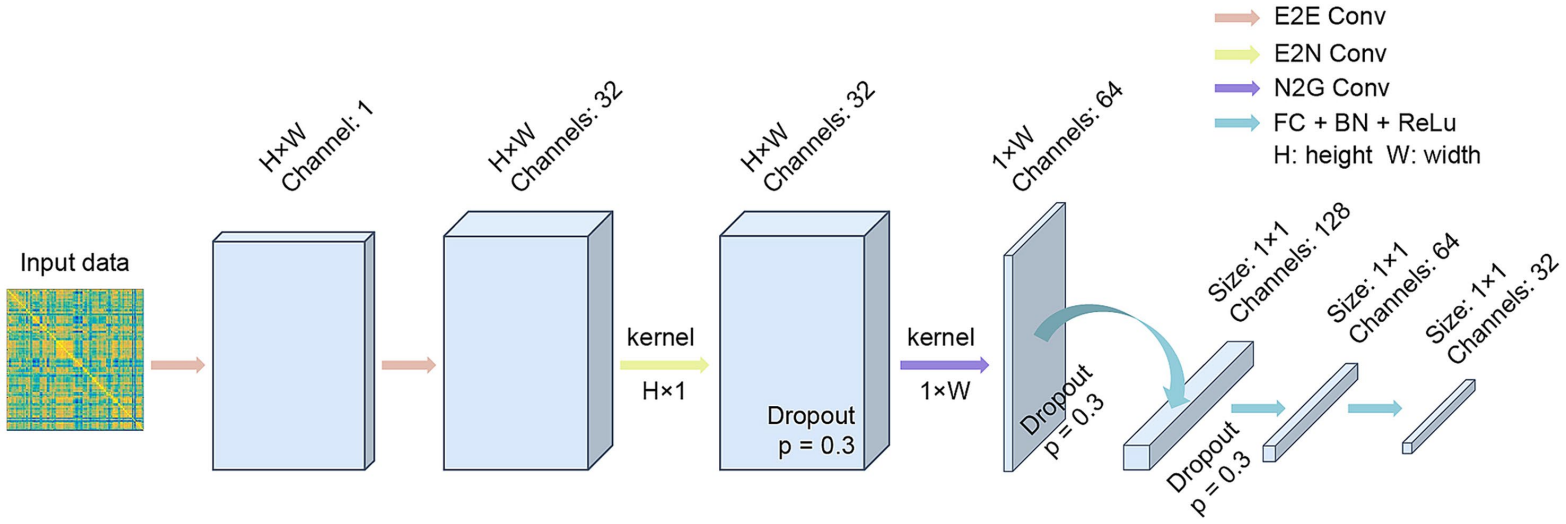
Schematic diagram of the BrainNet CNN architecture.

Based on the BrainNet CNN architecture (Figure 4), the dataset was randomly divided into a training set and an independent test set at a ratio of 8:2 (with 370 samples in the training set and 93 samples in the test set). Model development and hyperparameter tuning were performed exclusively on the training set using stratified five-fold cross-validation. Specifically, the training data were partitioned into five folds, with four folds (approximately 296 samples) used for model training and the remaining fold (approximately 74 samples) serving as an internal validation set during each iteration. To address class imbalance, the Synthetic Minority Over-sampling Technique was applied to the internal training subset, thereby balancing class distributions. The BrainNet CNN model was trained on the internal training subsets using the following hyperparameters: an initial learning rate of 1e−4, weight decay of 5 × 10^−4^, batch size of 8, and a maximum of 200 training epochs. A cosine annealing schedule was employed to dynamically adjust the learning rate throughout optimization. To prevent overfitting, an early stopping criterion was implemented based on validation loss; training was terminated if no meaningful improvement (defined as a decrease greater than 1 × 10^−4^) was observed for 20 consecutive epochs after the 70th epoch. A fixed random seed of 3,407 was used across all of the experiments to ensure reproducibility.

To evaluate the performance of our proposed architecture, we implemented four baseline models: Random Forest (RF), Support Vector Machine (SVM), Graph Convolutional Networks (GCN), and Graph Attention Networks (GAT). All models were developed using the same dataset, five-fold cross-validation split, and consistent input features as the BrainNet CNN experiments. For the traditional baselines, RF utilized 300–500 decision trees (depth 10–15), while SVM was optimized with RBF or linear kernels (C∈{0.1, 1, 10}). Both integrated a preprocessing flow of StandardScaler, feature reduction, and SMOTE. Concurrently, GCN and GAT models followed the exact experimental setting of our primary architecture to ensure architectural differences alone drive performance variations. To maintain rigor, the random seed was fixed at 3407, and class weights were balanced across all methods.

To further evaluate the model’s generalizability across diverse scanning environments and to mitigate potential confounding effects arising from multi-site data acquisition, we performed a Leave-One-Site-Out (LOSO) cross-validation. Specifically, the dataset was partitioned based on acquisition sites rather than a random split. In each iteration of the LOSO framework, the model was trained on data from all sites except one, which was held out entirely as an independent testing site to assess the model’s performance on a completely unseen scanning environment. Furthermore, as Sites 2 and 11 contained data from only a single diagnostic category, they were excluded from the analysis to prevent potential statistical bias and ensure a balanced multi-site comparison. To maintain rigorous methodological standards and prevent data leakage, data harmonization using the ComBat algorithm and oversampling via SMOTE were executed strictly within each training fold, ensuring that the test site remained entirely independent of the preprocessing and training pipelines.

### 2.6 Model performance evaluation and statistical analysis

An ensemble evaluation strategy was employed wherein the five best-performing models from the cross-validation procedure were applied to the independent test set to generate final predictions, thereby enabling a robust and comprehensive performance assessment. Core evaluation metrics, including area under the receiver operating characteristic curve (AUC), sensitivity, and specificity, were reported. To assess statistical reliability and model stability, 95% confidence intervals (CIs) and inter-fold variance were computed for each metric. Model calibration was evaluated using the Brier score and the Hosmer–Lemeshow (HL) goodness-of-fit test. Clinical utility was assessed via decision curve analysis (DCA), which estimated net benefit across a range of threshold probabilities. Additionally, training dynamics were visualized by plotting the mean accuracy and loss trajectories across both training and validation sets during the five-fold cross-validation.

Following the visualization of brain regions identified as relevant to classification, partial correlation analysis was conducted to examine associations between neuroimaging-derived measures and clinical variables, while controlling for potential demographic confounders. To account for multiple comparisons, all of the raw *p*-values were adjusted using the false discovery rate (FDR) method, with FDR-adjusted *p*-values < 0.05 considered to be statistically significant.

## 3 Results

### 3.1 Demographic and clinical characteristics

The final analysis included 111 males and 245 females with MDDSI. Detailed demographic and clinical characteristics across participating sites are summarized in Table 2. Baseline differences between the MDDSI and MDDNSI groups were assessed using two-sample t-tests for continuous variables and chi-square tests for categorical variables, as appropriate. Given the non-normal distribution of illness duration (in months) in both groups, the Mann–Whitney U test was used for between-group comparisons of this variable. The results revealed a statistically significant difference in sex distribution between the two groups.

**Table 2:** Demographic and clinical characteristics of participants across all of the sites.

Site	MDDSI			MDDNSI		
	Sample	Age	Sex (male/female)	Sample	Age	Sex (male/female)
Site 1	62	32.27 ± 8.30	22/40	11	29.64 ± 7.02	6/5
Site 2	30	43.87 ± 12.94	6/24	—	—	—
Site 6	11	32.55 ± 11.41	3/8	3	21.67 ± 3.21	2/1
Site 7	28	40.86 ± 11.39	9/19	8	47.75 ± 12.69	5/3
Site 8	30	31.97 ± 10.19	4/26	9	34.22 ± 8.60	4/5
Site 11	8	27.75 ± 11.37	3/5	—	—	—
Site 12	27	32.89 ± 9.29	5/22	5	43.40 ± 7.09	1/4
Site 14	42	31.50 ± 7.55	15/27	22	28.68 ± 5.84	6/16
Site 18	18	31.67 ± 6.79	4/14	3	30.00 ± 9.54	2/1
Site 19	19	38.63 ± 10.16	3/16	10	40.10 ± 13.15	1/9
Site 21	40	32.48 ± 12.85	17/23	24	37.79 ± 13.22	15/9
Site 22	22	33.70 ± 9.93	10/12	7	35.71 ± 12.27	4/3
Site 23	19	33.64 ± 9.37	10/9	5	26.60 ± 3.29	4/1
Total	356	33.83 ± 10.84	111/245	107	34.67 ± 11.54	50/57

### 3.2 Diagnostic performance of the proposed model

**Figure 5:**
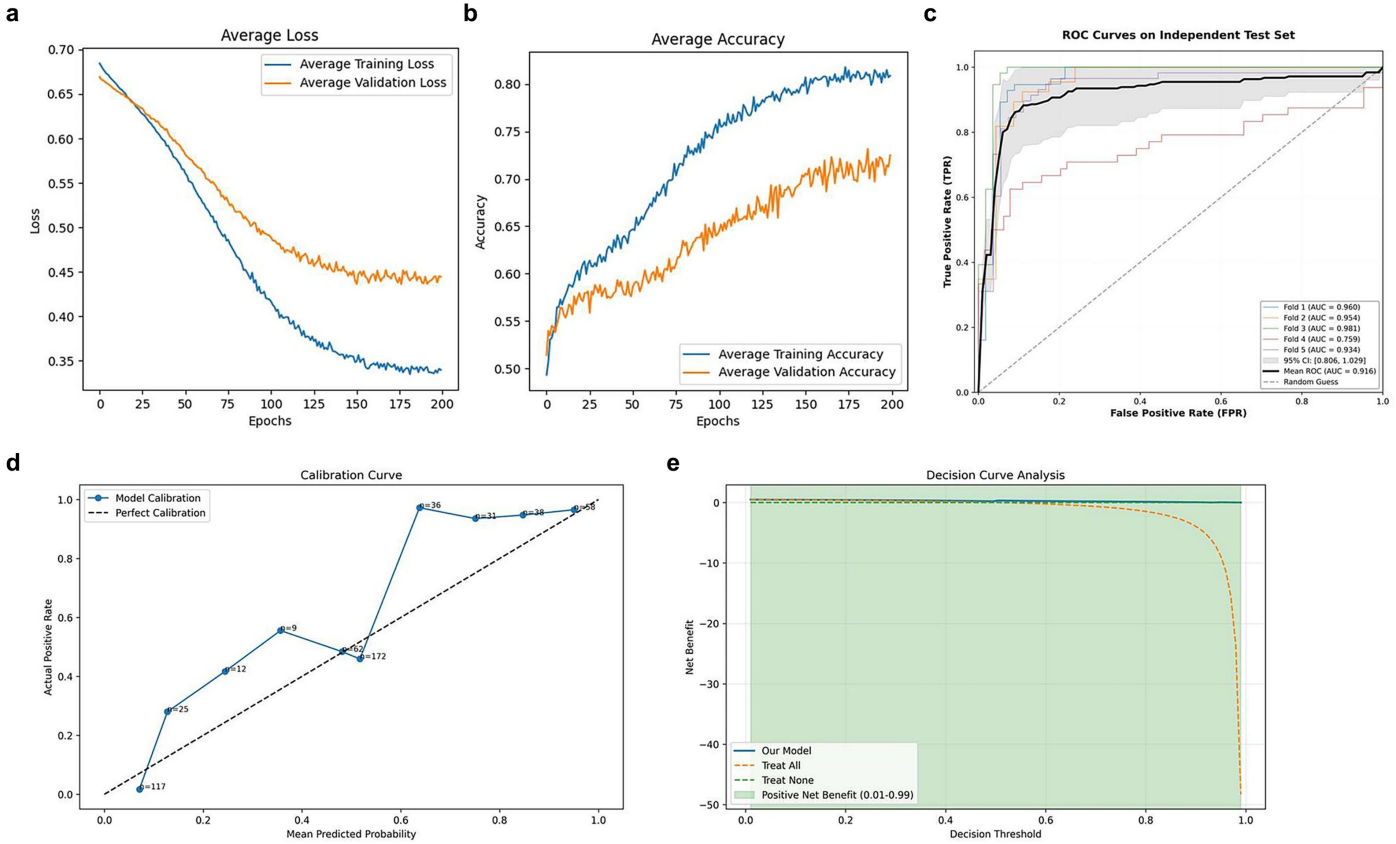
Model classification performance. **(a)** Comparison of average loss trajectories between training and validation sets. **(b)** Comparison of average accuracy trajectories between training and validation sets; **(c)** ROC curve for MDDSI prediction on the independent test set (95% confidence interval: [0.847–0.990]). **(d)** Calibration plot of predicted probabilities. **(e)** Decision curve analysis illustrating clinical net benefit across threshold probabilities.

During model training, the training loss decreased progressively with increasing epochs and converged to a low value of approximately 0.34. On the training set, the sensitivity, specificity, and AUC reached 96, 85%, and 0.95, respectively. In contrast, the validation loss initially decreased and subsequently fluctuated between 0.44 and 0.46 without exhibiting a consistent upward trend (Figure 5a). Regarding classification accuracy, training accuracy increased rapidly during the early epochs and stabilized at approximately 0.81, whereas validation accuracy improved in the early stages and then oscillated around 0.72 in later epochs (Figure 5b). Despite the observed performance gap between the training and validation sets, the stability of validation accuracy across epochs suggests that model generalization was maintained and that progressive overfitting did not occur.

On the independent test set, the model achieved a mean AUC of 0.916 (CI: 0.806–1.029; Figure 5c). The sensitivity and specificity were 0.952 and 0.851, respectively, with standard deviations of 0.025 and 0.180, indicating relatively low performance variability across different data subsets. Calibration analysis further demonstrated that higher predicted probabilities were generally aligned with higher observed event rates (Figure 5d). The calibration curve closely approximated the ideal diagonal, particularly within moderate probability ranges, with only minor deviations at the extremes. In line with this, the H–L goodness-of-fit test yielded a χ^2^ value of 10.870 with 8 degrees of freedom (*p* = 0.209), indicating no significant difference between predicted and observed outcomes and suggesting satisfactory model calibration. DCA demonstrated that, across a wide range of decision thresholds, the model yielded greater net clinical benefit compared to both “treat all” and “treat none” strategies, supporting its potential clinical utility (Figure 5e).

The experimental results of LOSO are summarized in Table 3. The model demonstrated robust and consistent performance across the majority of independent test sites. Specifically, high classification efficacy was observed in several centers, with Site 12 achieving a balanced accuracy of 0.91 and an AUC of 0.96, and Site 19 yielding a balanced accuracy of 0.92 and an AUC of 0.95. Other notable performances included Site 18 (AUC = 0.94) and Site 23 (AUC = 0.92). While some performance variability was noted in sites with smaller sample sizes or different clinical characteristics [Site 6 (AUC = 0.67) and Site 14 (AUC = 0.86 with lower sensitivity)], the overall performance across diverse scanning environments indicates that the model captured generalizable neurobiological features of suicidal ideation rather than site-specific noise.

**Table 3:** The experimental results of LOSO.

Test_Site	Balance_Acc	AUC	Spe	Sen
1	0.81	0.85	0.82	0.81
12	0.91	0.96	0.98	0.81
14	0.5	0.86	0.84	0.16
18	0.89	0.94	0.94	0.84
19	0.92	0.95	0.94	0.90
21	0.76	0.81	0.88	0.65
22	0.80	0.86	0.93	0.67
23	0.79	0.92	0.80	0.79
6	0.52	0.67	0.67	0.36
7	0.71	0.68	0.63	0.79
8	0.72	0.82	0.67	0.77

As summarized in Table 4, BrainNet CNN consistently outperformed all of the comparator models across evaluation metrics. Notably, state-of-the-art deep learning models such as GCN and GAT often struggle to generalize effectively when applied to small-scale, imbalanced datasets. Paired-sample *t*-tests conducted on AUC values across cross-validation folds confirmed that BrainNet CNN significantly outperformed all of the alternative models, thereby supporting the statistical robustness of its performance advantage.

**Table 4:** Performance comparison of baseline classifiers.

Model	Accuracy (%)	Sensitivity (%)	Specificity (%)	AUC (%)
GCN	50.06 ± 0.01	98.31 ± 0.05	1.82 ± 0.05	59.96 ± 0.13^✳^
GAT	52.03 ± 0.05	98.31 ± 0.05	1.82 ± 0.05	64.62 ± 0.05^✳^
RF	70.41 ± 3.13	79.19 ± 7.26	41.04 ± 12.63	70.70 ± 3.76^✳^
SVM	71.93 ± 2.47	76.39 ± 3.72	57.01 ± 9.93	72.35 ± 5.41^✳^
MLP_FC	85.43 ± 0.05	85.36 ± 0.09	85.50 ± 0.04	93.09 ± 0.03^✳ssssssw^
BrainNet CNN	88.22 ± 1.94	95.58 ± 2.51	85.07 ± 3.59	93.40 ± 1.67

An asterisk (*) indicates that the AUC of BrainNet CNN is statistically significantly different from that of the corresponding comparator model.

### 3.3 Visualization of discriminative brain regions

**Figure 6:**
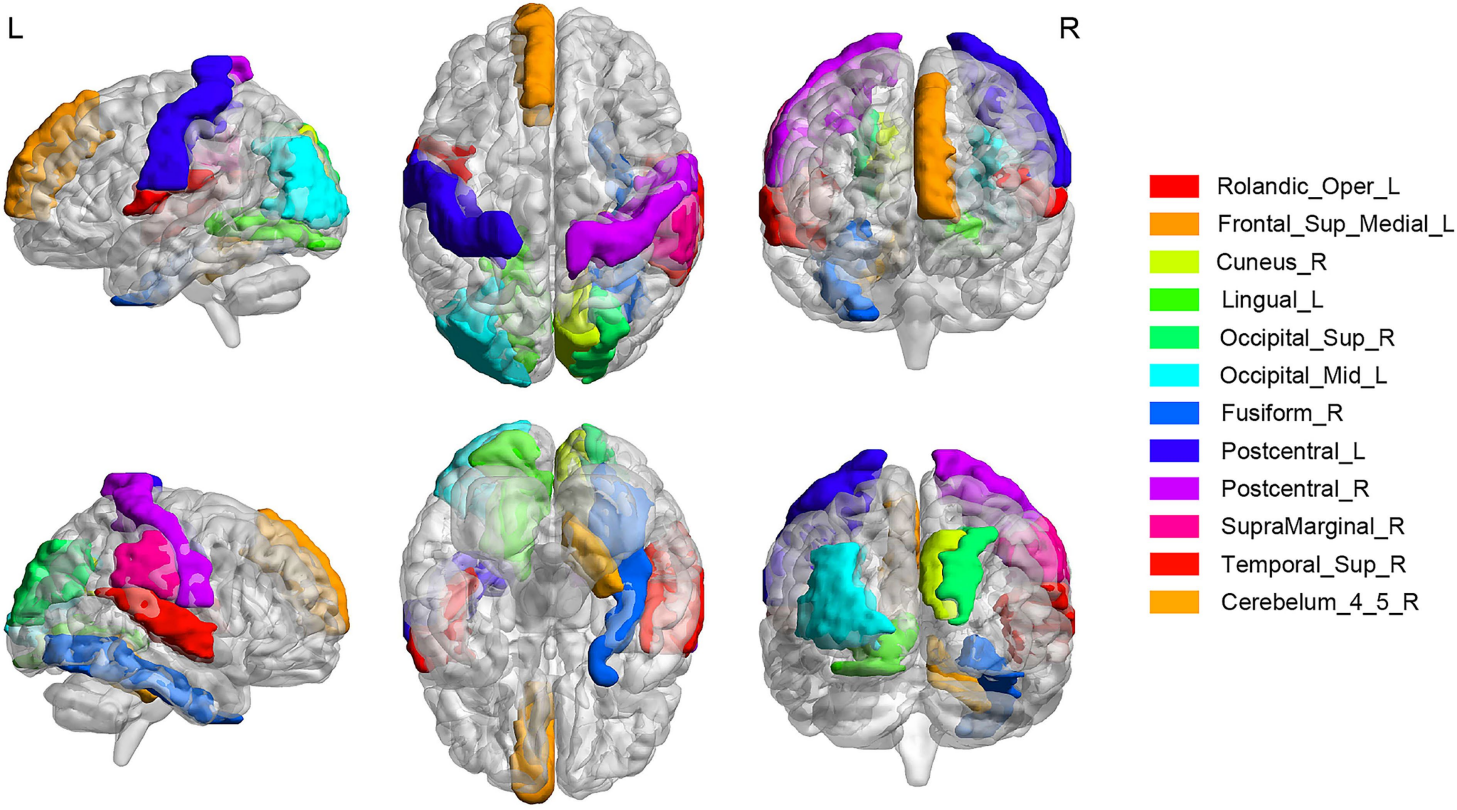
Visualization of brain regions contributing most significantly to the classification of MDDSI versus MDDNSI in the BrainNet CNN model. (L: Left hemisphere; R: Right hemisphere).

The brain regions identified as most contributory by the visualization analysis, along with their corresponding contribution scores, are presented in Table 5. These regions were primarily located in the rolandic operculum, medial superior frontal gyrus, cuneus, lingual gyrus, superior occipital gyrus, middle occipital gyrus, and cerebellum. A three-dimensional rendering of these regions is provided in Figure 6, illustrating their spatial distribution across the brain.

**Table 5:** Brain regions with the highest contribution to the classification of MDDSI versus MDDNSI in the BrainNet CNN model, along with their corresponding normalized contribution values.

AAL index	Brain region	Relative contribution
17	Rolandic_Oper_L	0.0305
23	Frontal_Sup_Medial_L	0.0298
46	Cuneus_R	0.0312
47	Lingual_L	0.6605
50	Occipital_Sup_R	0.0311
51	Occipital_Mid_L	0.0311
56	Fusiform_R	0.0308
57	Postcentral_L	0.0306
58	Postcentral_R	0.0312
64	SupraMarginal_R	0.0304
82	Temporal_Sup_R	0.0317
98	Cerebelum_4_5_R	0.0311

### 3.4 Associations between FC and SI severity

**Figure 7:**
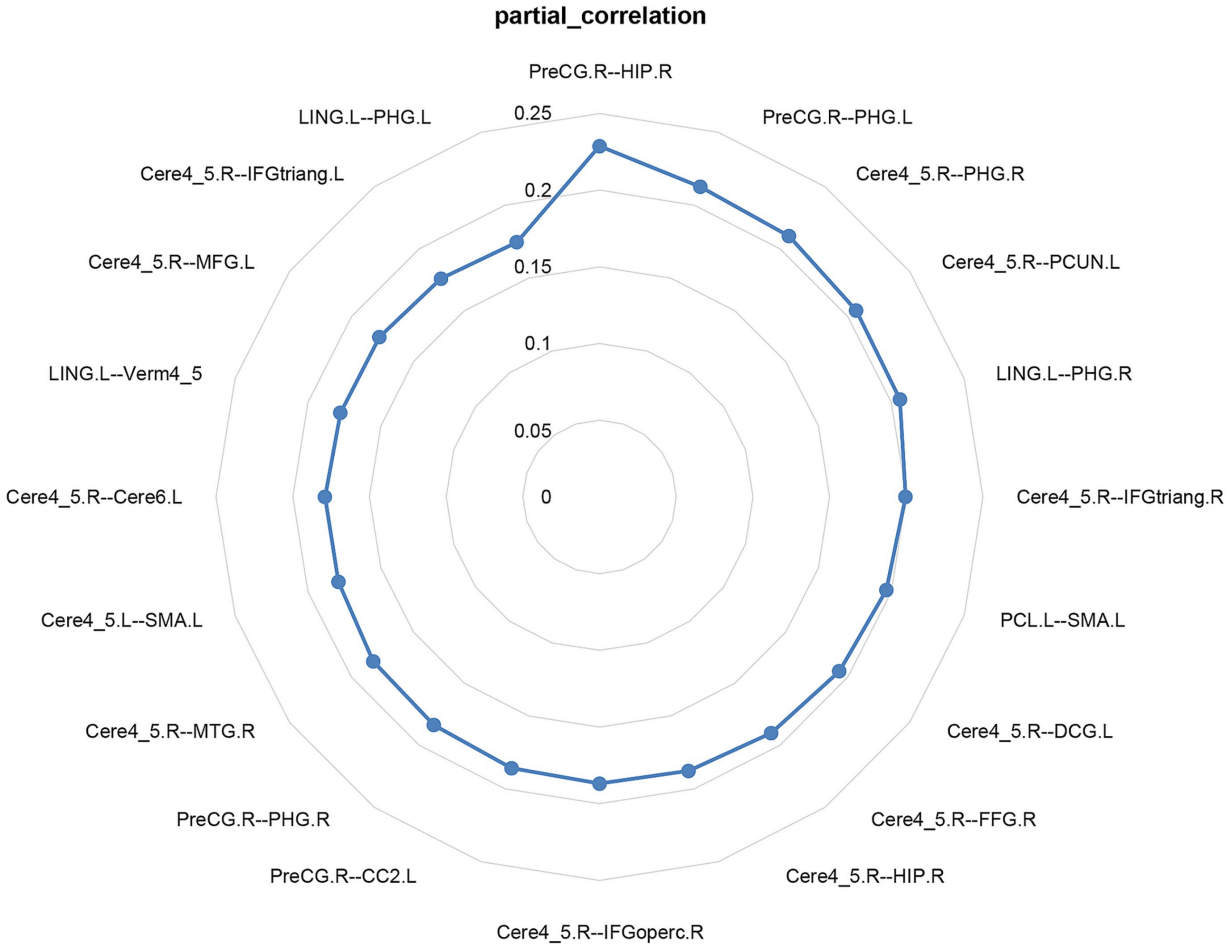
Radar plot of partial correlation coefficients between significant brain region FCs and HAMD suicide item scores. The plot displays the top 20 FCs with the strongest associations. FDR-corrected *p*-values < 0.05 were considered to be statistically significant.

After controlling for age and sex, partial correlation analyses were conducted to examine associations between candidate FC measures and scores on the suicide item of the HAMD. To evaluate the robustness of these associations to potential outliers and violations of normality assumptions, Huber robust regression was additionally performed to adjust for covariates and recompute partial correlation coefficients, yielding robust *p*-values (Table 6). Effect sizes were calculated by converting correlation coefficients (*r*) to Cohen’s d. Based on these analyses, the 20 FCs showing the strongest associations with suicide item scores were identified. These connections predominantly involved interactions between Grad-CAM–highlighted regions of interest and areas such as the hippocampus, parahippocampal gyrus, precuneus, frontal cortex, and cingulate gyrus. These connections demonstrated statistically significant partial correlations with HAMD suicide item scores after FDR correction for multiple comparisons (Table 6; Figure 7). Moreover, within the MDDSI subgroup, stronger FC values were positively correlated with suicide item scores, suggesting that increased connectivity strength was associated with greater SI severity.

**Table 6:** Statistical summary of the top 20 FCs showing the highest partial correlations with SI severity.

Functional connectivity	Spearman’s ρ	95% CI	*P_FDR*	Robust_ρ
Temporal_Sup-Hippocampus	0.2513	[0.151, 0.346]	0.0002	0.2489
Temporal_Sup- Putamen	0.2380	[0.137, 0.334]	0.0003	0.2355
Cerebelum_4_5_R-ParaHippocampal	0.2287	[0.128, 0.323]	0.0015	0.2291
Fusiform-Precuneus_L	0.2228	[0.122, 0.319]	0.0013	0.2223
Fusiform-Cerebelum_Crus1_R	0.2249	[0.124, 0.321]	0.0013	0.2241
Cerebelum_4_5_R-Hippocampus	0.2122	[0.111, 0.309]	0.0017	0.2122
Cerebelum_4_5_R- Frontal_Inf_Tri	0.2101	[0.108, 0.307]	0.0017	0.2071
Cuneus -ParaHippocampal	0.2087	[0.107, 0.306]	0.0083	0.2090
Cerebelum_4_5_R- Cingulum_Mid	0.2084	[0.107, 0.306]	0.0017	0.2069
Cerebelum_4_5_R-Precuneus	0.2082	[0.107, 0.306]	0.0017	0.2079
Fusiform-Precuneus_R	0.2075	[0.106, 0.305]	0.0023	0.2075
Postcentral-Hippocampus	0.2073	[0.106, 0.305]	0.0054	0.2053
Fusiform- Supp_Motor_Area	0.2049	[0.103, 0.302]	0.0023	0.2039
Fusiform- Cingulum_Mid	0.2008	[0.099, 0.298]	0.0024	0.1990
Cerebelum_4_5_R-Fusiform	0.1946	[0.093, 0.293]	0.0002	0.1940
Occipital_Sup - Cingulum_Mid	0.1907	[0.089, 0.289]	0.0173	0.1891
Lingual-ParaHippocampal	0.1830	[0.081, 0.282]	0.0387	0.1817
Cerebelum_4_5_R- Frontal_Inf_Oper	0.1870	[0.085, 0.285]	0.0041	0.1844
Rolandic_Oper - Frontal_Inf_Tri	0.1841	[0.082, 0.283]	0.0159	0.1821
Fusiform- Occipital_Inf	0.1761	[0.074, 0.275]	0.0059	0.1751

Includes correlation coefficients, FDR-adjusted *p*-values, robust *p*-values from Huber regression, and corresponding effect sizes.

## 4 Discussion

The World Health Organization has identified MDD as the leading risk factor for suicidal behavior, accounting for approximately 56%–87% of all suicide cases globally (Rihmer, 2007). Moreover, epidemiological data indicate that 10%–15% of individuals diagnosed with MDD eventually die by suicide (Liu et al., 2020). Despite this substantial risk, the clinical identification of MDDSI continues to rely primarily on clinicians’ subjective judgment and rating scale assessments. These approaches are highly dependent on clinical experience, potentially limiting diagnostic accuracy and delaying timely intervention. Accumulating evidence suggests that patients with MDDSI exhibit dysregulated FC in neural circuits involved in emotion, cognition, and behavioral regulation (Chen et al., 2024; Dobbertin et al., 2023; Wen et al., 2025). However, findings across studies remain inconsistent, which may reflect methodological limitations and the pronounced heterogeneity of both MDD and SI. As a result, the neurobiological mechanisms underlying MDDSI remain insufficiently characterized.

To address these limitations, the present study employed resting-state fMRI and a deep learning framework to identify patients with MDDSI and investigate potential neuroimaging biomarkers. Using fMRI data from MDD patients, whole-brain FC features were derived based on the AAL atlas, and BrainNet CNN—a convolutional neural network designed to capture brain network topology—was applied to classify MDDSI. The proposed framework demonstrated strong discriminative performance in distinguishing patients with SI from those without. In comparison, one prior study used a neural network approach incorporating demographic, clinical, and biological variables to identify SI in MDD patients, achieving 70.08% accuracy (Ge et al., 2020), while another employed a support vector machine using dynamic FC features, reporting an accuracy of approximately 75% (Xu et al., 2022). In contrast, the current model achieved an accuracy of 88%, with a sensitivity of 95%, specificity of 85%, and an AUC of 0.93 on an independent test set, indicating a notable improvement over previous machine learning models.

In addition to classification, this study applied BrainNet CNN in combination with Grad-CAM to visualize and interpret discriminative neuroimaging features from a large-scale, multicenter dataset. The brain regions contributing most significantly to classification were not randomly distributed but were primarily located within the DMN, salience network (SN), visual processing network, and cerebellum. This widespread distribution suggests that MDDSI is unlikely to result from dysfunction in a single region but instead reflects altered integration across multiple neural systems.

In the feature visualization analysis, the left lingual gyrus demonstrated a normalized contribution value an order of magnitude greater than that of any other identified region, suggesting that aberrant FC involving this structure may represent the most discriminative neuroimaging signature for differentiating MDDSI from those without. In MDDSI, lingual gyrus abnormalities may reflect a pathological bias toward negative visual imagery, whereby death-related or threatening visual content is spontaneously and intrusively rehearsed. This process is further amplified through the lingual gyrus’s coupling with the parahippocampal gyrus and hippocampus. Consistent with this, the present study found that Lingual_L–ParaHippocampal functional connectivity was significantly associated with HAMD suicide item scores (*r* = 0.2060, FDR-corrected *p* = 0.0104). Furthermore, increased FC between the lingual gyrus and visual recognition areas — including the middle occipital gyrus and fusiform gyrus — has been reported in depressed patients with SI (Deng et al., 2025), and multimodal meta-analytic evidence has further demonstrated that functional correlations among the lingual gyrus, fusiform gyrus, and middle occipital gyrus are jointly disrupted in MDD with suicidality, indicating coordinated dysregulation within the occipital visual network (Lin et al., 2025).

Notably, the superior medial frontal gyrus—a key hub of the DMN—was highly influential in classification, potentially reflecting abnormal DMN function related to pathological rumination and increased self-referential processing in depression. The SN, particularly the rolandic operculum (insula), also showed high importance, potentially indicating network dysfunction related to the processing of internal distress in MDDSI. Furthermore, the involvement of visual processing regions, such as the occipital lobe and fusiform gyrus, supports emerging evidence of perceptual biases in mood disorders. Prior studies have reported associations between FC involving the fusiform gyrus and SI scores on the HAMD (Gao et al., 2023; Zhong et al., 2024), suggesting that visual processing abnormalities may modulate how external stimuli are integrated with internal emotional states, contributing to SI.

*Post hoc* correlation analyses also revealed significant associations between cerebellar FC and HAMD suicide scores, particularly involving connections with cortical and subcortical structures, including the frontal and temporal lobes, precuneus, parahippocampal gyrus, and limbic regions. These findings underscore the cerebellum’s role in differentiating SI within MDD populations. In addition to its role in motor coordination, the cerebellum is increasingly recognized as a contributor to emotional regulation and cognitive control via cerebello-cerebral circuits. The current findings align with those of (Deng et al., 2025), who reported reduced cerebellar FC with DMN-related regions (e.g., temporal gyri, precuneus, and inferior parietal lobule) and increased FC with visual recognition areas (e.g., lingual gyrus, middle occipital gyrus, and fusiform gyrus) in patients with depression. Importantly, the observed associations between cerebellar connectivity and higher-order cortical networks—particularly the DMN and limbic system—may reflect disrupted cerebellar modulation of emotion-processing circuits that is specifically linked to SI.

This study has several limitations. First, the cross-sectional design limits the generalizability of the findings; future longitudinal studies are warranted to validate and expand upon the observed results. Second, although the ROI–based approach adopted here is effective for identifying key neural signatures in limited patient samples, it may not fully capture all of the brain regions potentially implicated in suicidality. Third, suicidal ideation (SI) was classified based solely on a single item (Item 3) from the HAMD-17. As the reviewer rightly noted, this single-item approach lacks clinical granularity and does not capture the severity dimensions of suicidality, which may have introduced misclassification bias and limited our ability to examine dose-dependent relationships between neural alterations and suicidal thinking. Furthermore, given the substantial heterogeneity in both MDD and suicidal behavior, future studies should examine the influence of additional variables—such as psychiatric comorbidities, illness duration, and age of onset—to enhance classification accuracy and improve the clinical applicability of neuroimaging-based diagnostic models.

In summary, this study developed an interpretable deep learning framework based on resting-state fMRI for the auxiliary diagnosis of MDDSI. Leveraging a large multicenter dataset and multiple validation strategies, the BrainNet CNN model demonstrated robust classification performance. The integration of Grad-CAM enabled the identification and visualization of brain regions contributing most significantly to model predictions, thereby enhancing neurobiological interpretability.

The results revealed prominent alterations in FC involving the cerebellum, DMN, and visual processing networks. These connectivity patterns were further associated with SI severity, as measured by scores on the suicide item of the HAMD. Collectively, these findings introduce a novel methodological framework for applying explainable artificial intelligence techniques to the identification of neuroimaging biomarkers in psychiatric disorders and underscore the potential of interpretable deep learning models to support the objective assessment of suicide risk in patients with MDD.

## Data Availability

The original contributions presented in the study are included in the article/supplementary material, further inquiries can be directed to the corresponding author.

## Footnotes

1
http://www.rfmri.org/maps
